# VIGOROUS PHYSICAL ACTIVITY, DEPRESSION, PRO-INFLAMMATORY CYTOKINE AND RACIAL/ETHNIC VARIATIONS IN FALLS

**DOI:** 10.1093/geroni/igad104.1980

**Published:** 2023-12-21

**Authors:** Ayse Malatyali, Tom Cidav, Rui Xie, Ladda Thiamwong

**Affiliations:** University of Central Florida, Orlando, Florida, United States; The Johns Hopkins University School of Medicine, Baltimore, Maryland, United States; University of Central Florida, Orlando, Florida, United States; University of Central Florida, Orlando, Florida, United States

## Abstract

Physical activity plays a key role in preventing falls among older adults as it supports muscle strength and improves balance and postural control. Recent studies have revealed that physical activity modulates Interleukin-6 (IL-6) levels and reduces the incidence of falls among older adults. This study describes the relationships between vigorous physical activity, IL-6 plasma levels, depression, and falls among older adults. We conducted a cross-sectional study on Health and Retirement Study participants aged 65 and above (n= 9,942) using datasets from the 2016 interviews. Of the sample, 37.7% reported falling in the last 24 months. Mean ages were: 77.4 (8.14) years in participants who had a fall and 75 (7.31) years in participants without a fall. Participants engaging in regular vigorous physical activity were 24% less likely to have a fall than those not engaging in vigorous activity. We quartiled the IL-6 levels and presented quartile-4 as the most elevated IL-6 levels. Compared to quartile-1 (lowest), participants in quartile-3 were 24%, and those in quartile-4 were 45% more likely to fall. The effect of IL-6 was insignificant in quartile-2. Having depression was also significantly associated with falls (OR=2.40). We observed a significant inverse relationship between ethnic/racial minorities and odds of falling: Hispanics were 16%, and African Americans were 36% less likely to fall than non-Hispanic whites. The likelihood of falling among people living in rural was significantly higher than among those living in urban (OR= 1.15). Interleukin-6 may be incorporated to fall prevention interventions as an indicator of fall risk.

